# Small Extracellular Vesicles (sEV) in Surgical Drain Fluids of Oral Squamous Cell Carcinoma Patients Carry Luminal and Surface DNA

**DOI:** 10.3390/ijms27104577

**Published:** 2026-05-20

**Authors:** Alicja Gluszko, Zhongping Xu, James F. Conway, Monika Pietrowska, Adel Chaudhuri, Jose Zevallos, Theresa L. Whiteside

**Affiliations:** 1 Department of Pathology, University of Pittsburgh School of Medicine and UPMC Hillman Cancer Center, Pittsburgh, PA 15213, USA; gluszkoa@upmc.edu; 2 Department of Biochemistry, Medical University of Warsaw, 02-091 Warsaw, Poland; 3 Department of Otolaryngology Head and Neck Surgery, University of Pittsburgh School of Medicine and UPMC Hillman Cancer Center, Pittsburgh, PA 15213, USA; zhx81@pitt.edu (Z.X.); zevallosjp@upmc.edu (J.Z.); 4 Department of Structural Biology, University of Pittsburgh School of Medicine and Pittsburgh Center for Cryo-EM, Pittsburgh, PA 15213, USA; jxc100@pitt.edu; 5 Maria Sklodowska-Curie National Research Institute of Oncology, 44-100 Gliwice, Poland; monika.pietrowska@gliwice.nio.gov.pl; 6 Institute of Genetics and Animal Biotechnology, Polish Academy of Sciences, ul. Postepu 36A, Jastrzebiec, 05-552 Magdalenka, Poland; 7 Department of Radiation Oncology, Mayo Clinic, Rochester, NY 55902, USA; chaudhuri.aadel@mayo.edu

**Keywords:** small extracellular vesicles (EVs), exosomes, surgical drain fluid (SDF), vesicular DNA, oral squamous cell carcinoma (OSCC)

## Abstract

Postoperative surgical draining fluid (SDF) in oral squamous cell carcinoma (OSCC) was reported to contain circulating free DNA and extracellular vesicles (EVs). The potential role of SDF DNA as a cancer biomarker in OSCC patients is investigated. To determine whether EVs contribute to the DNA content of SDF, small EVs (sEVs) were isolated by size exclusion chromatography and compared with those in paired plasma. sEV obtained from a cohort of HPV(+) and HPV(−) patients were evaluated for the endocytic origin, size, vesicular morphology, and the DNA content. The sEV from HPV(+) and HPV(−) SDF were similar. The sEVs from SDF were larger and contained more DNA than the sEV from plasma. Treatments of sEV with the DNase/RNase cocktail established the presence of DNA on the surface and in the sEV lumen. Furthermore, 20 to 30% of the total SDF DNA was associated with sEV. Fragmentomic analyses identified the largest DNA (>10,000 bp) on the vesicle surface. The DNA in the vesicle lumen consisted of smaller (~5000 bp) DNA fragments. Electron microscopy of the enzyme-treated vesicles indicated that surface DNA/RNA may be involved in maintaining vesicular integrity and modulating the vesicle entry into cells. The sEV-carrying surface and luminal DNA emerge as significant contributors to the total SDF DNA and, following validation, might serve as useful biomarkers of disease presence/progression in OSCC.

## 1. Introduction

Extracellular vesicles (EVs) are highly heterogeneous, cell-derived, membrane-bound vesicles present in all body fluids, including blood, urine, saliva, breast milk, cerebrospinal fluid, ascitic fluid, and surgical drain fluids [1,2,3]. Based on their distinct sizes and biogenesis pathways, EVs can be classified into small EVs (sEVs) or exosomes, microvesicles (MVs), and apoptotic bodies [3]. sEVs are the most widely studied EV subset because of their origin in the endosomal compartment of parental cells, where they are packaged with molecular and genetic cargo resembling that of their parent cell [4]. Upon release into the extracellular space and then into the peripheral circulation [5,6], sEV transports molecular contents, including proteins, lipids, nucleic acids, and metabolites, to nearby or distant sites, serving as an intercellular transportation/communication network [2]. Their molecular/genetic cargo is encapsulated by a phospholipid-bilayer membrane and, thus, is protected from degradation in the circulation [7]. sEVs are involved in the regulation of diverse biological processes, including immune modulation, cell differentiation, cellular senescence, and tissue repair/regeneration [8,9,10].

sEVs are produced under both physiologically normal and pathological conditions. sEV may acquire the pathogenic capability from their parent cells to spread/exacerbate diseases and have been implicated in the pathogenesis of a wide range of diseases, including cancer [11,12,13,14]. Specifically, tumor cell-derived sEVs, called TEX, regulate tumor development and progression, promote metastasis formation, and modulate immune cell functions [9]. Emerging data indicate that sEV, especially TEX carrying a cargo resembling tumor cells, may be ideal cancer biomarkers [12,15]. Compared to circulating tumor cells (CTCs) and circulating tumor DNA (ctDNA), TEX have unique advantages as liquid tumor biopsy: (i) they are proactively and selectively secreted by tumor cells; (ii) they are released in excess by tumors, and they are abundant in body fluids; (iii) their molecular/genetic contents are protected by the lipid bilayer membrane; (iv) TEX interact with and are readily taken up by healthy and malignant cells; and (v) TEX induce functional changes in recipient cells, which can be measured and correlated to disease progression/regression [4,5,15,16].

In cancer, circulating free (cf)DNA and ctDNA have recently been evaluated as promising biomarkers of disease progression/response to therapy [17,18]. Specifically, we have studied cfDNA in surgical draining fluid (SDF) and paired plasma of patients with locally advanced oral squamous cell carcinoma (OSCC) who underwent neck dissection [19]. We reported that cfDNA in SDF had greater sensitivity than plasma for quantitative detection of postoperative minimal residual disease (MRD), correlated with pathology, and predicted disease recurrence [19]. Others reported that molecular and metabolic profiling of sEV in OSCC, specifically sEV isolated from postsurgical drainage fluids, positively correlate with lymph node metastasis [20,21]. However, none of these studies compared cfDNA vs. sEV as potential biomarkers of metastatic disease. This may be due to the widely held perception that sEVs contain little or no DNA, making the comparison impossible. However, several recent publications convincingly demonstrated the presence of DNA with the size and mutational status of cellular DNA in sEV, suggesting that genomic DNA is detectable in the vesicle lumen [22,23,24,25,26].

In this study, we address the issue of DNA presence/abundance in sEV isolated from SDF and paired the plasma of HPV(+) and HPV(−) patients with OSCC. We report that relative to plasma, SDF is enriched in sEV, which carry DNA on the cell surface and in their lumen. Our data suggest that sEV carrying non-fragmented genomic DNA as well as surface-associated cfDNA, which are enriched in SDF, and may be of special value as potential biomarkers of OSCC progression, USA (Gibco, Grand Island, NY, USA) (Thermofisher, Pittsburgh, PA, USA), 2024.

## 2. Results

### 2.1. Characterization of sEV Isolated from SDF and Plasma

**Figure 1:**
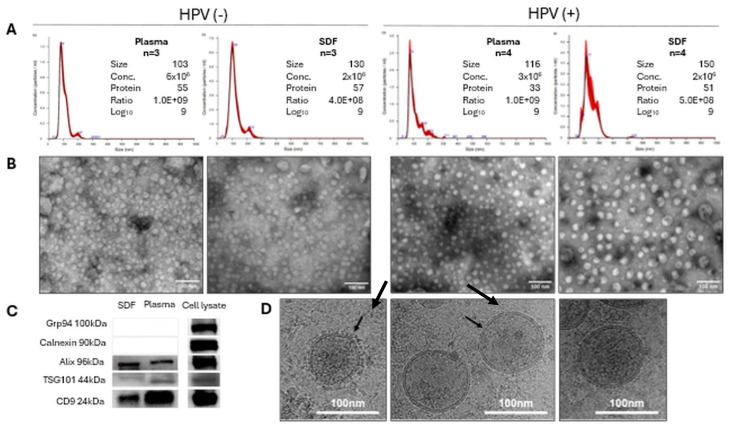
Characteristics of sEV isolated from SDF and paired plasma of patients with OSCC. (**A**,**B**) represent the NTA results and corresponding TEM images of sEV isolated from paired plasma and SDF specimens of HPV(−) and HPV(+) patients. The inserted data in (**A**) are mean values for three HPV(−) and four HPV(+) patients, respectively, normalized to one mL fluid for: (i) the particle size in nm; (ii) the particle number/mL; (iii) the protein concentration in µg/mL; (iv) the ratio of particle#/protein µg; and (v) the Log10 ratio of particle#/protein µg. (**C**) western blots of sEV isolated from paired SDF and plasma. Each lane was loaded with 5 µg protein. (**D**) shows the Cryo-EM images of representative sEV isolated from SDF, illustrating sEV surrounded by a membrane, various vesicle sizes, and the presence of loosely structured “corona” on the outer surface of the vesicular membranes (see arrows).

The sEV were isolated from paired SDF and plasma specimens of seven patients with OSCC [four HPV(+) and three HPV(−)], and were evaluated for the protein content, vesicle morphology (TEM, Cryo-EM), vesicle size and numbers (NTA), the ratio of vesicle number/vesicle protein conc., and the selected protein cargo by Western blots. All data were normalized to 1 mL of plasma or SDF (Figure 1A–D). It is important to stress that SDF was collected on the first or second day of surgery, and thus closely reflected the vesicular content of the tissues surrounding the tumor. The vesicles from SDF were larger than those from paired plasma, with a mean size ranging from 140 nm to 110 nm, respectively. The particle numbers were lower in SDF than in the plasma, with mean numbers ranging from 2 × 10^10^ to 4.5 × 10^10^ particles/mL, respectively. The protein concentration was also higher in SDF (54 µg/mL) relative to plasma (44 µg/mL). The ratio of sEV #/µg protein was somewhat lower for EVs in SDF than plasma, although the log10 ratio values were approximately the same (Log10 = 9), suggesting a similar level of vesicle “purity.” Western blots showed that sEVs contained CD9, TSG101, and ALIX, but did not carry cytoplasmic proteins, Grp94, or Calnexin (Figure 1C), confirming their endosomal origin. Cryo-electron microscopy showed that each sEV was surrounded by a double membrane faintly decorated by a “corona” of surface molecules (Figure 1D). In agreement with ISEV criteria for the EVs nomenclature, the vesicles were classified as small EVs (sEV) [27].

### 2.2. Circulating Free (cf)DNA and sEV-Associated EV-DNA in SDF vs. Plasma

**Figure 2:**
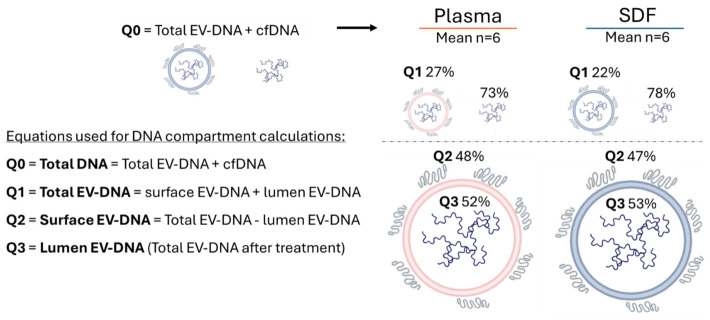
The DNA content in the body fluids of patients with OSCC. Based on the QuBit analysis, plasma contained 70 ng/mL of the total DNA, and SDF contained 150 mg/mL of the total DNA (mean of six assessments for each fluid; see Table 1).

The SDF and plasma of patients with HNSCC were previously reported to contain cfDNA [19]. To evaluate the possibility that some of the cfDNA in SDF and plasma was associated with sEV, we measured the total DNA (i.e., before sEV isolation) in SDF and paired plasma samples by QuBit. We then isolated sEV from all the SDF and plasma samples and measured the DNA extracted from the vesicles by QuBit. In both SDF and plasma specimens, sEV-associated DNA comprised around 25% of the total DNA (Figure 2). However, while plasma contained only 70 ng/mL of the total DNA (cfDNA + EV-DNA), SDF contained 150 mg/mL of the total DNA. Thus, while in SDF and plasma, the distribution of cfDNA and EV-DNA was almost equal, SDF contained a ~3000-fold higher abundance of total DNA than paired plasma. This finding indicates that SDF represents a rich source of cfDNA, as well as EV-DNA for further studies.

Table 1 shows the raw data for DNA quantifications by QuBit [ng/mL] used to calculate the mean and percentage values of the total DNA in plasma or SDF and DNA associated with the surface and the lumen of EVs isolated from the body fluids, as indicated in Figure 2.

### 2.3. Optimization of the Vesicle Treatment with DNase/RNase

**Figure 3:**
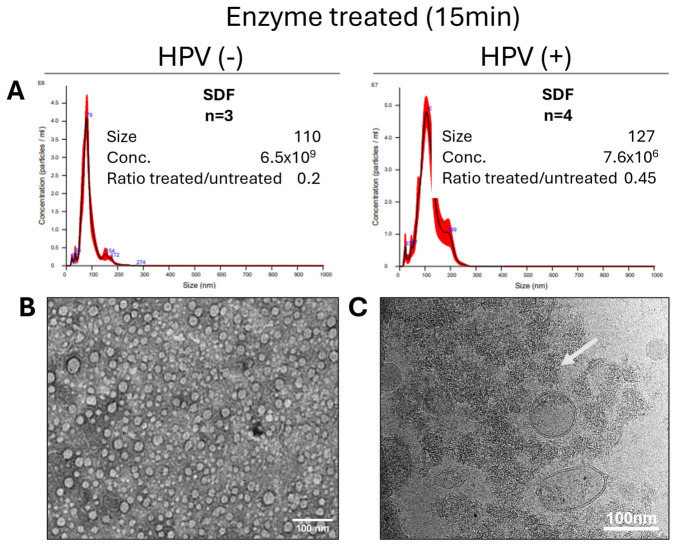
Results of 15 min treatments of sEV isolated from SDF of OSCC patients with the DNase/RNase cocktail. (**A**) shows the NTA for HPV(−) and HPV(+) samples treated with the enzyme cocktail for 15 min at RT. The data for the sEV size and concentration (particle number/mL fluid) are mean values of seven experiments. The ratio “treated/untreated” reflects EV concentration before and after enzyme treatments, and values below zero indicate a loss of vesicles and a decrease in their numbers and size. (**B**) shows a TEM image of sEV treated with the DNase/RNase cocktail for 15 min. (**C**) shows Cryo-EM images of the enzyme-treated (15 min) vesicles and illustrates the presence of numerous vesicle “ghosts”, indicated by an arrow.

To determine whether EV-DNA is present on the vesicle surface or in the vesicle lumen, we treated sEV isolated from SDF with a cocktail of DNase/RNase, as described in Methods. Initially, 15 min enzymatic treatments were performed using sEV isolated from plasma and SDF of the seven patients with OSCC. The NTA results from these experiments, presented in Figure 3A, show that the 15 min treatment of isolated sEVs, with the enzyme cocktail containing 0.05 U/μL of DNase plus 0.05 µg/µL RNase at RT, resulted in a decreased sEV size and numbers relative to untreated sEV (compare Figure 3A with Figure 1A). Electron microscopy of the treated sEVs (Figure 3B,C) confirmed that treated vesicles were a heterogeneous mix of very small vesicles and vesicle “ghosts.”

Thus, 15 min or longer enzymatic treatments led to vesicle fragmentation and a loss of the sEV integrity.

**Figure 4:**
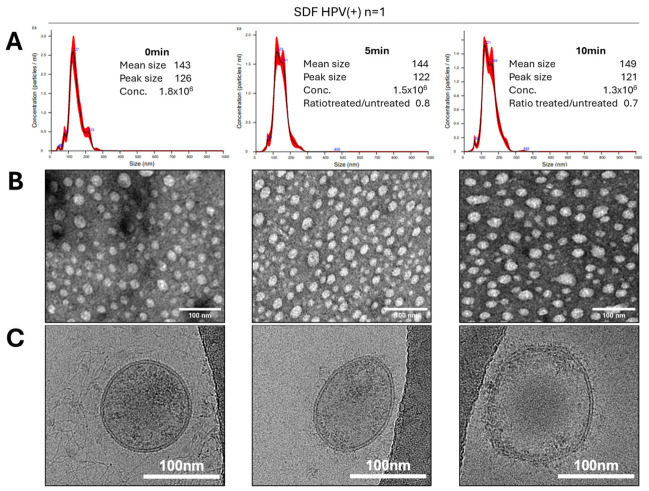
Effects of short-term treatments of sEV with the DNAse/RNase cocktail. Representative data obtained with sEV isolated from an SDF specimen (patient #269 HPV(+)) treated for 0, 5, and 10 min with the cocktail. (**A**) shows the NTA results. (**B**) shows the TEM images. (**C**) shows the selected Cryo-EM images of untreated and enzyme-treated EVs.

These results suggested that excessive enzymatic treatments might destroy sEV. Since our objective was the removal of sEV surface-associated nucleic acids without compromising vesicle integrity, the optimization of treatment conditions was necessary. Therefore, sEVs isolated from the SDF of an HPV(+) patient were treated with the same enzyme cocktail of DNase/RNase for 5 or 10 min and tested for alterations in vesicle size, numbers, morphology, and ability to interact with recipient cells in vitro. Figure 4 shows that while the 5 min treatment did not disrupt the vesicles or changed their shape, the peak size of treated vesicles decreased, while their concentration/mL also decreased slightly. After the 10 min treatment, the peak size and sEV concentration/mL were basically unchanged, while the mean size increased (probably because of the aggregate formation). TEM clearly indicated that at 5 min, the bulk of treated vesicles appeared smaller than that of untreated vesicles, although by Cryo-EM, the vesicular morphology and double membrane surrounding each vesicle remained intact. In contrast, at 10 min, TEM indicated a partial distortion of the vesicular morphology in numerous vesicles, many of which appear to be smaller in size and misshapen, but still were surrounded by a double membrane, as confirmed by Cryo-EM.

**Figure 5:**
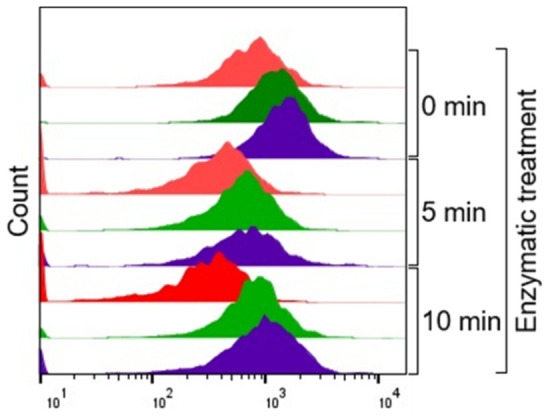
Effects of treatment of sEV on the time-related entry of sEV into Jurkat T cells. sEV labeled with the vFRed membrane stain were either treated or not treated with the DNase/RNase cocktail for 5 or 10 min and co-incubated with recipient Jurkat T cells. sEV internalization after 15 min (red), 30 min (green), or 60 min (purple). The co-incubation was measured by flow cytometry. Untreated sEV (0 min treatment) served as a positive control for the vesicle uptake.

To determine whether the DNase/RNase treatment interfered with sEV functions, we measured the uptake of sEV by recipient Jurkat T cells. sEVs labeled with a fluorescent dye were either treated or not treated with the DNase/RNase cocktail and then examined for vesicle entry into the recipient cells using flow cytometry. Figure 5 shows that after 15 min co-incubation of Jurkat cells with the enzyme-treated sEV (red), the vesicle entry was delayed relative to the entry of non-treated sEV. After 30 min of co-incubation (green), the entry was still delayed, but by 60 min (purple), it was almost equal to the untreated controls, especially for vesicles treated with the enzyme cocktail for 10 min. In another uptake experiment using sEVs labeled with the PKH-26 dye, 96–99% of Jurkat cells contained untreated stained sEVs at 15, 30, and 60 min compared to 89–99% for vesicles treated with the enzymes for 5 min and 98–99% for vesicles treated for 10 min. In aggregate, these data suggest that the presence of nucleic acids on the surface of sEV may play a role in their initial contact with recipient cells. However, the initial delay in the sEV entry seen at 15 min with vesicles pre-treated for 5 min with enzymes was no longer seen with the sEV pre-treated for 10 min. This suggests that the gradual removal of nucleic acids from the corona leads to subtle structural surface changes in receptor-ligands responsible for sEV internalization, which may be transient and do not significantly impair the vesicle entry. This speculation is supported by the results of the apoptosis experiments. As previously reported, the sEV from the plasma of OSCC patients induces apoptosis of Jurkat T cells [28]. However, when we tested the ability of the enzymatically treated sEV to induce apoptosis of Jurkat T cells, we found it was comparable to apoptosis induced by untreated sEV. These results indicated that the subtle structural changes on the vesicle surface induced by the enzyme cocktail had no effect on the ability of these vesicles to induce apoptosis of recipient cells. As previously reported, sEV-induced apoptosis of Jurkat cells is only partly dependent on external death receptor signaling and is a result of external stress leading to intrinsic apoptosis [29]. Once delivered to the cytosol of the recipient cells, enzymatically treated sEVs were as effective in inducing mitochondrial dysfunction, and the resulting cell deaths were similar to the non-treated sEVs.

### 2.4. Evidence for DNA Presence on the Surface and in the Lumen of EVs

**Figure 6:**
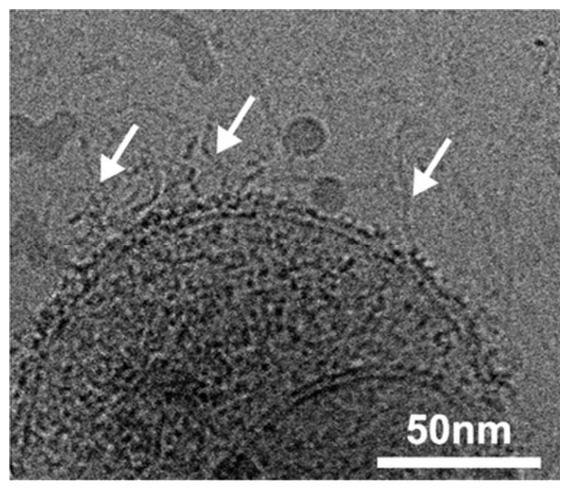
Cryo-EM of a representative individual sEV from SDF, illustrating the presence of “strands” surrounding the outer surface of vesicular double membrane (see arrows).

An examination of the sEV isolated from SDF by Cryo-EM showed vesicles ranging in size between 30 and 200 nm, surrounded by a double membrane and a corona of densely packed material (Figure 6). The vesicle surface was loosely encircled by “strands” that were evident in the background of untreated sEV. Following the 5 min treatment with the DNase/RNase cocktail, the “strands” disappeared, suggesting that nucleic acids formed their backbone. Images of sEV treated with DNase/RNase for 5 min consistently showed vesicles that were largely intact but no longer surrounded by a ‘corona” or “strands”.

Next, we extracted DNA from the untreated and enzyme-treated (5 min) sEVs isolated from the SDF of patients with HPV(−) and HPV(+) OSCC. As described above and illustrated in Figure 2, we determined the total DNA in SDF consisting of cfDNA plus total EV-DNA. After sEV was isolated from SDF, the vesicle surface-bound DNA was removed after 5 min treatment with DNase/RNase, and the luminal DNA was extracted from the vesicles and quantified by Qubit. The levels of sEV-surface-associated DNA were calculated by subtracting the luminal sEV DNA from the total sEV DNA (see Figure 2 and Table 1). This revealed that about 47% of DNA associated with sEV was located on the vesicle surface, and ~53% represented luminal DNA.

**Figure 7:**
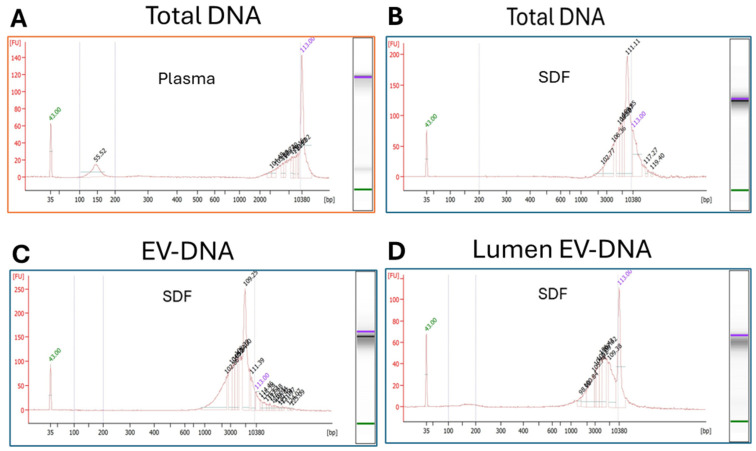
High Sensitivity Bioanalyzer data of DNA from paired plasma and SDF samples from a representative HPV(+) patient. (**A**,**B**) show the Bioanalyzer data for the total DNA (cfDNA + EV-DNA) in plasma and SDF. (**C**) shows the data for EV-DNA in SDF. (**D**) shows the data for DNA in the EV lumen. The X-axis = [bp] and the Y-axis = [fluorescence signal].

The results of the QuBit and High Sensitivity Bioanalyzer experiments indicated that sEV-associated DNA is localized on the vesicle surface (47%), as well as in the vesicular lumen (53%). As shown in Figure 7A, the total DNA in plasma contained small DNA fragments, plus larger fragments [2000 to 10,000 bp], while the total DNA in the paired SDF contained mostly large DNA fragments (2000 to >10,000 bp). Because the concentration of DNA in the sEV from plasma was very low and not sufficient even for High Sensitivity Bioanalyzer tests, only SDF samples were analyzed for the presence of EV-DNA. In Figure 7C, EV-DNA consists mainly of the large DNA fragments (2000 to 10,000 bp) and the very large DNA fragments (>10,000 bp). In contrast, the DNA in the lumen of sEV (Figure 7D) consists of larger fragments (2000 to 10,000 bp) minus the very large fragments. These results suggest that the largest DNA fragments are localized to the sEV surface, while the large DNA fragments are in the sEV lumen.

## 3. Discussion

Postoperative drainage fluid (PDF), defined as the fluid exudate from the wound site following lymph node dissection, has been recognized as a rich source of biological components, including proteins, nucleic acids, EVs, cytokines, cells, and bacteria [30]. PDF is a mixed exudate of blood and lymph, and its composition likely reflects real-time changes in the patients’ postoperative status, potentially serving as a liquid tumor biopsy [30]. PDF, also referred to as “Surgical draining fluid (SDF)”, has been previously evaluated as a source of circulating tumor DNA (ctDNA) [19] and as a source of EVs in patients with OSCC [21]. Conceptually, it appears likely that draining the tissues immediately surrounding the surgically removed tumor wouldprovide fluids enriched in ctDNA and/or in TEX that could be useful in biomarker discovery studies. Accordingly, our group has recently reported that tumor-informed cfDNA and cell-free HPV-DNA quantified in SDF from patients with OSCC correlated with the high TNM stage, extranodal extension (ENE), and high-risk clinic-pathological features of the disease [19]. These results indicated that ctDNA in SDF can potentially serve as a promising biomarker of MRD in OSCC [19]. In another study evaluating sEV in SDF and aimed at defining their surfactome and proteomic profiles, Qu et al. [20] found a highly significant overexpression of several immuno-modulatory proteins in sEV, including CD24, CD44, CD63, and CD146. Differential expression levels of these proteins in EVs isolated from SDF, but not from patients’ plasma, suggested that the EVs in SDF reflected the tumor-microenvironment (TME) and, by mediating local immune suppression, contributed to cancer progression. It also suggested that EVs in SDF could be better biomarkers of disease than EVs present in the patients’ peripheral circulation. In still another study, Wang and colleagues [21] studied the proteomic profiles of EVs isolated from PDF in LN(+) and LN(−) patients with OSCC, and reported that EVs in PDF were mainly derived from epithelial cells and immune cells. They identified several differentially overexpressed proteins in EVs of patients with LN(+) OSCC, suggesting that the protein cargo of EVs in PDF correlated with the presence of LN metastasis [21].

To date, no studies of EV-associated nucleic acids have been reported for EVs isolated from SDF in patients with OSCC. This may reflect the generally held view that mRNA, miRNA, and DNA, although detectable in EVs, are present in low abundance, making their recovery and analysis difficult [31]. For many years, the presence of DNA in circulating EVs has been a subject of considerable controversy [32,33,34,35]. Based on earlier reports [36], an impression that DNA is detectable only in larger microvesicles, which contain more nucleic acids than sEV (exosomes), has persisted in the field. It is only recently, as methods for the EV isolation and their characterization have significantly improved, that the successful nucleic acid extraction from sEV has become routine. This led to the emergence of convincing evidence for the presence in the tumor-derived EVs of double-stranded DNA with the mutational profile identical to that of the tumor [23,24,35,36,37].

With this background in mind, we proceeded to evaluate the contribution of sEV to the total DNA in SDF and paired plasma specimens. It is important to emphasize that when body fluids are tested for DNA, these fluids invariably contain sEV. Thus, sEV-associated DNA likely contributes to the total DNA in SDF. Interestingly, the total DNA recovered from an equivalent fluid volume was significantly greater (~3000-fold) in SDF than in paired plasma. While sEVs isolated from SDF were larger and more heterogeneous in size than plasma sEVs, their numbers were lower, suggesting that fewer but larger sEVs in SDF may contain more DNA. Also, sEV-associated DNA in SDF, especially DNA in the vesicle “corona,” appeared to be less fragmented than cfDNA. The presence of DNA on the sEV surface may be important for stabilizing vesicular morphology and for sEV interactions with cells. The removal of surface DNA from sEV appeared to slow down sEV’s entry into the cytosol of the recipient cells, but did not interfere with their uptake and functional consequences of entry, such as the death of the recipient’s T cells [29]. This agrees with our previously reported data that sEV’s entry into the recipient’s T cells only partly involves death-receptor ligands on the vesicle surface and is largely dependent on the stress-inducing signaling events that translate into mitochondrial dysfunction and intrinsic T cell apoptosis [29].

About 50% of sEV-associated DNA was localized to the vesicular lumen. Protected by the vesicle membrane, fragments of luminal DNA were not affected by exo-enzymes. This suggests that luminal DNA might be a better liquid biopsy biomarker than cfDNA. Similar distribution of large DNA fragments between the sEV surface and lumen was previously reported by Chetty et al. [22] for sEVs isolated from leukemia cell lines. Several other studies of sEVs isolated from cancer cell lines or body fluids of cancer patients report on the presence in the vesicle lumen of large DNA fragments that carry mutations seen in parent tumor cells [35,37]. In view of these reports, the role of the DNA presence in sEV as a biomarker of cancer detection and progression is significantly enhanced. Clearly, the accessibility of genomic-mutated DNA in sEV facilitates a liquid biopsy analysis without the dependence of a “tumor informed” cfDNA approach. Further, in patients with HPV(+) OSCC, it is reasonable to expect that the vesicular components might include viral proteins and/or DNA. In our earlier proteomic study (unpublished data), the MS/MS scans of viral peptides in HPV(+) OSCC-derived sEV identified three viral peptides derived from three different HPV proteins: a minor capsid protein E2, probable protein E5, and replication protein E1. We confirmed by confocal immuno-microscopy that HPV(+) sEV contained viral E2 proteins, while HPV(−) sEV did not. Recently, using digital droplet PCR (ddPCR), we confirmed the presence of HPV E6 DNA in sEV isolated from SDF of an HPV(+) patient (unpublished data). These preliminary data indicate that assessments of the HPV status of sEV would further solidify their role as diagnostic/prognostic biomarkers in OSCC.

This study of SDF-derived sEV has several limitations. This was a small “proof-of-principle” study that provided translationally significant insights about sEV as potential cancer biomarkers, but did not allow for clinical correlations. The levels of vesicular DNAs or of fragment size in the lumen DNA were not different in sEV obtained from the SDF of HPV(+) vs. HPV(−) OSCC. The study was not designed to perform sequencing for tumor-specific mutations in the lumen DNA or for HPV integration, which would be necessary for the meaningful evaluation of sEV as OSCC biomarkers. With the small patient cohort, only one clinically relevant comment can be made. In one of four HPV(+) patients, whose cancer recurred and who died 16 weeks after surgery, sEV contained the highest level of total and vesicular dsDNA. As HPV(+) patients generally have a more favorable prognosis than those with HPV(−) disease, this example suggests that examining sEV in post-surgery SDF might have prognostic significance. A study with a larger cohort of OSCC patients and a more extensive evaluation of sEV in SDF, including DNA sequencing for tumor mutations and HPV integration, is currently in progress at our institution.

## 4. Materials and Methods

### 4.1. Cell Line

The Jurkat T cell line expressing surface CD8 protein was obtained from Dr. H. Rabinowich (Department of Pathology, University of Pittsburgh, Pittsburgh, PA, USA) and cultured in a RPMI medium, supplemented with a 10% (*v*/*v*) exosome-depleted and heat-inactivated fetal bovine serum (FBS, Gibco, Grand Island, NY, USA), 100 U/mL penicillin, and 100 ug/mL streptomycin, at 37 °C and in an atmosphere of 5% CO_2_ in air. CD8+Jurkat cells were periodically cultured in the selection medium (RPMI plus Geneticin, ThermoFisher, Waltham, MA, USA). Prior to experiments, Jurkat cells were tested for the expression of surface CD8 using flow cytometry, and 70–80% of Jurkat cells were CD8+. Cell cultures were routinely tested for Mycoplasma (Lonza Mycoplasma detection kit, ThermoFisher, USA) and were negative.

### 4.2. Patient Samples

Paired plasma and surgical drain fluid (SDF) specimens from OSCC patients (*n* = 4 HPV(+) and n = 3 HPV(−)) were collected as per the IRB-approved protocol #99-069. The study included patients undergoing surgical neck dissection with histologically confirmed OSCC. Pathological staging information, including lymph node status, was obtained to confirm the presence/absence of lymph node metastasis. Table S1 presents the clinico-pathological data of the patients, including stage, recurrence, and survival. All patients signed the IRB-approved consent forms for specimen collection and the use of personal data for research. The collected specimens were annotated, and each was given a unique label and processed for EV isolation as described below. Paired plasma specimens were collected preoperatively on the day of surgery.

The SDF was collected on the first and second days after the surgery from the patients who received neck dissection. Samples were obtained from routinely used post-operative drainage bags in the ward. Drainage fluid was collected over 24–48 h at room temperature using a closed collection system to avoid contamination. After transferring the drainage fluid to the laboratory, the SDF samples were supplemented with a low concentration cocktail of protease inhibitors (Santa Cruz Biotechnology, Dallas, TX, USA). Then, the samples were centrifuged at 500× *g* for 10 min and 2000× *g* for 15 min at 4 °C to deplete cells and cell debris. The SDF samples were aliquoted into 2 mL vials and stored at −80 °C. Samples were thawed immediately prior to sEV isolation. Only one freeze–thaw cycle was allowed for each sample in preparation for EV isolation.

### 4.3. sEV Isolation and Characterization

Thawed plasma and SDF paired samples were pre-cleared by sequential low-speed centrifugation, followed by centrifugation at 12,000× *g* for 30 min at 4 °C to sediment microvesicles (MVs). Pellets containing MVs were recovered and cryopreserved, while supernatants were harvested, ultra-filtered using bacterial 0.22 µm filters, and prepared for sEV isolation by size exclusion chromatography (SEC), eluting vesicles with PBS as previously described [38]. Fraction #4 containing the bulk of isolated sEV in PBS was concentrated using 100,000 MWCO Vivaspin 500 centrifugal concentrators (Sartorius Corp., Bohemia, NY, USA). The sEV protein concentrations were measured using a BCA protein assay kit (Pierce Biotechnology, Rockford, IL, USA) as directed by the manufacturer.

Transmission electron microscopy (TEM) of vesicles in the SEC fraction #4 was performed at the Center for Biologic Imaging at the University of Pittsburgh. The vesicles were placed on copper grids, stained with 1% (*v*/*v*) of uranyl acetate in ddH_2_O, and were visualized using TEM (model JOEL JEM-1011). Cryo-electron microscopy was performed by Dr. James Conway at the Center for Structural Biology, University of Pittsburgh.

The concentration and size distribution of sEV or TEX were measured using a nanoparticle tracking analysis (NTA) using NanoSight 300 (Malvern Panalytical, UK). The captured videos were analyzed using NTA software (2016), maintaining the screen gain and the detection threshold at 1 and 5, respectively. To determine the mean particle size/concentration in each sample, five consecutive measurements were obtained and averaged.

Western blots were used to evaluate the sEV or TEX protein profiles. Vesicle aliquots (5 µg protein) were lysed with a Laemmli sample buffer (Bio-Rad Laboratories, Hercules, CA, USA), separated using 12% SDS/PAGE gels, and after transferring from the gel to the polyvinylidene fluoride (PVDF) membranes, the proteins were detected using Abs specific for antigens, carried by sEV (Alix #MA5-32773, TSG101 #PA5-31260, Invitrogen and CD9 #13174, Cell Signaling) or antigens absent in sEV (Grp94 #20292 and Calnexin #2679, Cell Signaling, Danvers, MA, USA).

### 4.4. DNase/RNase Treatment

DNase I (ThermoFisher Scientific, Waltham, MA, USA, EN0521), an endonuclease that digests single- and double-stranded DNA, was used to determine the surface or lumen localization of DNA in vesicles. Aliquots of freshly isolated sEV (from Fraction #4; 10^10^ particles), suspended in reaction buffer (PBS + MgCl_2_), were incubated with a cocktail of DNase I and RNase A (ThermoFisher Scientific, Waltham, MA, USA, EN0531) at the final concentration of 0.05 U/µL and 0.05 µg/µL, respectively, for 5, 10, or 15 min at room temperature (RT). Reaction was stopped by adding 50 mM of EDTA in a volume equal to that of MgCl_2_ reaction buffer and RiboLock RNase Inhibitor (ThermoFisher Scientific, Waltham, MA, USA, EO0381) at a 1:1 ratio to RNase A.

### 4.5. DNA Isolation, Quantification, and DNA Fragment Analyses

DNA was extracted from the pre- and post-enzymatically treated sEV using the PureLink Genomic DNA Mini Kit (K1820-00), according to the manufacturer’s instructions. Samples were eluted twice with 25 µL of elution buffer. DNA was quantified by QuBit analysis, and a High Sensitivity DNA assay was used for fragment analysis (Bioanalyzer, Santa Clara, CA, USA), both performed at the Cancer Genomics Facility, UPMC Hillman Cancer Center, University of Pittsburgh.

### 4.6. Morphologic, Phenotypic, and Functional Changes in DNase/RNase Treated sEV

To test whether DNase/RNase treatment interfered with sEV characteristics and functions, several types of assays were performed. First, transmission electron microscope (TEM) and Cryo-EM images of sEV treated and untreated with the enzymes were obtained to evaluate the vesicular integrity. Next, the size and concentrations of enzyme- treated and untreated sEVs were determined by NTA. Phenotypic surface profiles of sEV before and after DNase/RNase treatment were studied by on-bead flow cytometry as previously described [39]. Uptake of labeled tumor-derived sEV by recipient Jurkat cells was evaluated. sEVs were labeled with the membrane dye (vFRed; #CBS4A, Cellarcus Bioscience, La Jolla, CA, USA) according to the manufacturer‘s instructions. Labeled sEVs were treated with DNase for 0, 5, or 10 min as described above. Aliquots (10 ug protein) of treated or untreated sEV were co-incubated with Jurkat T cells (1 × 10^5^/well) for 0, 15, 30, or 60 min. Uptake of labeled sEVs by Jurkat T cells and sEV presence in Jurkat cytosol were monitored by flow cytometry (Cytoflex, Beckman Coulter). The ability of sEV to induce Jurkat T cell apoptosis was measured by flow cytometry using the Apoptosis Annexin V kit (Invitrogen, Carlsbad, CA, USA, V13025), as previously described by us [29]. Briefly, Jurkat T cells (1 × 10^5^/well) were co-incubated with increasing concentrations of DNase-treated or untreated sEV (conc. range 2.5, 5.0, 10 ug protein) for 6 h, and their apoptosis was expressed as % of dead Jurkat cells in the gate.

### 4.7. Statistical Analysis

Statistical analyses were performed using GraphPad Prism 8.3 software (GraphPad Software Inc, La Jolla, CA, USA). A paired Student’s t-test was used to compare the independent samples of two patient groups (plasma-derived vs. SDF-derived EVs). A non-paired t-test was used to compare samples from HPV(+) vs HPV(−) samples. The data are presented as means ± SD of three independent experiments. The study also included 3 groups of EV samples: not treated, treated for 5 min, and treated for 10 min with enzymes. The differences between EVs’ size, numbers, and protein concentrations were measured by t-test and, for multivariate comparisons, by one-way Anova, followed by a Mann−Whitney test. *p*-values < 0.05 were considered significant.

## 5. Conclusions

Small extracellular vesicles (sEVs) in the SDF of OSCC patients carry luminal and surface DNA. The sEVs in SDF are enriched in DNA relative to sEVs in paired plasma. DNA associated with the vesicle “corona” consists of large DNase/RNase-sensitive DNA fragments, which support vesicular integrity and modulate the vesicle entry into recipient cells. About 50% of sEV-associated DNA is localized to the vesicular lumen and is protected from enzymatic degradation by DNase/RNase. The levels of vesicular DNA or of the fragment size in the lumen DNA were not different in the sEV obtained from the SDF of HPV(+) vs. HPV(−) OSCC patients. sEVs carrying surface and luminal DNA are significant contributors to the total SDF DNA, and could serve as potentially useful disease biomarkers following further validation in a larger patient cohort.

## Figures and Tables

**Table 1:** Raw data for DNA quantifications by QuBit [ng/mL].

	Plasma			SDF		
**Sample ID**	**Q0 Total DNA** (cfDNA + EV-DNA)	**Q1 EV-DNA** (no DNase cocktail)	**Q3 Lumen EV-DNA** (EV-DNA + DNase cocktail)	**Q0 Total DNA** (cfDNA + EV-DNA)	**Q1 EV-DNA** (no DNase cocktail)	**Q3 Lumen EV-DNA** (EV-DNA + DNase cocktail)
DF017 HPV (+)	40.8	14.8	5	98,800	10,800	11,300
DF025 HPV (+)	146	16.2	10.4	216,000	51,000	26,000
DF051 HPV (+)	44.5	24	10.8	97,200	24,400	10,400
DF127 HPV (−)	55	31.8	14.6	98,400	22,300	13,500
DF107 HPV (−)	14.2	8.3	5.8	157,600	37,300	17,900
DF090 HPV (−)	118	17.6	12.4	228,000	54,000	27,700
Mean	70	19	10	149,333	33,300	17,800
%Q1	100	27		100	22	
%Q3		100	52		100	53

## Data Availability

The data supporting the conclusions of this article are available upon request to the corresponding author.

## Supplementary Materials

The following supporting information can be downloaded at: https://www.mdpi.com/article/10.3390/ijms27104577/s1.
