# Supplementary material for: Small Extracellular Vesicles (sEV) in Surgical Drain Fluids of Oral Squamous Cell Carcinoma Patients Carry Luminal and Surface DNA
Source: Int J Mol Sci. 2026 May 20;27(10):4577. doi: 10.3390/ijms27104577 (PMC13206980; doi:10.3390/ijms27104577)
Supplement: Supplementary file 1 [file ijms-27-04577-s001.zip › ijms-4291824-supplementary.pdf]

**Table S1:** Clinico-pathologic Data of Patients

| | HPV Status | Sex | Age | Site of Primary | T | N | M | ENE | Recurrence | OS(mos) |
|---|---|---|---|---|---|---|---|---|---|---|
| 1 | NEG | F | 61 | Buccal | 2 | 2 | YES | YES | NO | 22 |
| 2 | NEG | F | 54 | Tongue | 1 | 0 | NO | NO | YES | 22 |
| 3 | NEG | F | 42 | Tongue | 3 | 0 | NO | NO | NO | 22 |
| 4 | P16 IHC | M | 57 | UNK | 0 | 1 | YES | NO | NO | 26 |
| 5 | P16 IHC | M | 51 | BOT | 4 | 2 | YES | YES | YES | DECEASED |
| 6 | P16 IHC | M | 73 | Tonsil | 1 | 1 | YES | YES | NO | 18 |
| 7 | P16 IHC | M | 55 | Tonsil | 1 | 1 | YES | NO | NO | 26 |
